# Colour Stability of 3D-Printed and Prefabricated Denture Teeth after Immersion in Different Colouring Agents—An In Vitro Study

**DOI:** 10.3390/polym14153125

**Published:** 2022-07-31

**Authors:** Mariya Dimitrova, Bozhana Chuchulska, Stefan Zlatev, Rada Kazakova

**Affiliations:** 1 Department of Prosthetic Dental Medicine, Faculty of Dental Medicine, Medical University of Plovdiv, 4000 Plovdiv, Bulgaria; bogana_68@abv.bg (B.C.); stefan.zlatev@mu-plovdiv.bg (S.Z.); rada.kazakova@mu-plovdiv.bg (R.K.); 2 CAD/CAM Center of Dental Medicine, Research Institute, Medical University of Plovdiv, 4000 Plovdiv, Bulgaria

**Keywords:** 3D printing, colour stability, denture teeth, dental resin, removable dentures

## Abstract

This study investigated the colour stability of three dimensional (3D)-printed and conventional denture teeth after immersion in different colourants. A total of 60 artificial maxillary central incisors were selected from three types of materials: 3D-printed dental resin (NextDent, 3D Systems, Soesterberg, The Netherlands), prefabricated acrylic teeth in Ivostar Shade (Ivoclar Vivadent, Schaan, Liechtenstein), and SpofaDent Plus in shade A2 (SpofaDental, Jičín, Czechia). These were immersed in four types of colourants at room temperature (23 °C ± 1 °C), including artificial saliva (pH = 6.8) as a control group, coffee, red wine, and Coca-Cola (*n* = 5). The temperature and the pH of the colouring agents were maintained throughout all immersion periods. After 7 days (T1), 14 days (T2), and 21 days (T3), the ∆E values were measured with a SpectroShade Micro (SpectroShade, Oxnard, CA, USA) spectrophotometer. Their means were then calculated and compared by two-way ANOVA. The independent factors, immersion time and different staining solutions, as well as the interaction between these factors, significantly influenced ΔE. The highest and the lowest mean ∆Es were recorded for prefabricated teeth in red wine, and 3D-printed teeth in artificial saliva, respectively. All the specimens demonstrated an increased colour change at T1 compared to T3, and the difference in mean ∆E was statistically significant.

## 1. Introduction

Patients with edentulism have a condition indicating prosthetic treatment with complete removable dentures. Dental resins, differing in composition and processing technology, are currently used in prosthodontics for these types of prosthetic restorations. All resins have a satisfactory aesthetic and functional effect in the oral cavity [1]. Despite the number of advantages, they also have some distinct disadvantages—their volumetric and optical characteristics change over time [2].

The consumption of different foods and beverages and the imbibition of water lead to changes in the colour stability of the dentures and their aesthetic qualities. Complete removable dentures must function well, which means they must be comfortable, durable, stable, and have a good aesthetic appearance [3,4].

The main disadvantages of the denture resins used in the fabrication of complete removable dentures are that their aesthetic, physical, and mechanical properties change rapidly over time in the oral cavity [5]. For aesthetics, colour stability is one of the criteria that needs attention. The appearance and colour of the artificial teeth are important properties of the removable denture. In addition, denture teeth should match the shade and shape of the anthropometric characteristics of the patient [6]. Changes in the physical and mechanical properties of dental materials cause irreversible defects and lead to the material’s aging [7]. Poor oral hygiene and frequent consumption of some beverages are the main reasons for the staining of the denture resins. Certain beverages, such as coffee, red wine, and tea, have been shown to influence the colour stability of dental materials used in removable prosthodontics [8].

The Munsell colour system determinates colours based on three properties: hue, chroma, and value [9]. This system is used in the visual identification of tooth shade. Colours are fully specified by listing the three hue, value, and chroma numbers, in that order.

The system of the Commission of the Internationale de l’Eclairage (CIE) L * a * b * is an international organization for colour standardization, which determines the amount of acceptable change in the colour of each experimental body and includes three coordinates: L *, a *, and *b* * [10].

L *—refers to bleaching, whose value varies from 0 for perfect black to 100 for perfect white.

a *—the coordinates of the chromaticity in the red–green axis.

b *—the coordinates of the chromaticity in the yellow–blue axis.

The CIELAB system is the most diffused system, but CIEDE2000 (∆*E00*) is preferred because it is more sensitive to appreciable changes [3]. It is a modification of CIELAB and gives an overall best performance in predicting experimental datasets. The typical applications are pass or fail decision, colour constancy, metamerism, and colour rendering [4]. The CIELAB system is used in the objective and precise determination of colour in instrumental measurement. First, the differences in the whitening coefficients are defined by chromaticity (Δ*L*, Δ*a*, Δ*b*) as a result of exposure to ultraviolet rays. The total colour change (Δ*E ab*) can be determined using the following formula (Equation (1)):

(1) $$\Delta E_{ab}^{*}=\sqrt{\Delta L^{*2}+\Delta a^{*2}+\Delta b^{*2}}$$

The main shade of the tooth is presented in the middle third, and the range of colours varies from incisal to gingival area [11]. Determining the colour of the teeth can be carried out in two main ways: visually in a clinical setting or instrumental with the help of equipment—a colorimeter, spectrophotometer, and digital photo analysis. Visual thresholds are of paramount importance as a quality control tool; they guide the evaluation and selection of dental materials and their clinical performance [12]. However, high inconsistency is observed in the visual determination of colours.

Instrumental determination is highly recommended as an additional method in everyday dental practice. There are various devices for determining the colour, measuring the entire tooth surface, creating a colour map, and measuring the arithmetic mean of the colour of a certain area (3–5 mm) of the tooth surface [13]. They decrease the subjective influence of the operator and the need for special lighting conditions. Spectrophotometers are the most accurate devices for colour analysis, but they are relatively expensive and more complicated to use. They measure the intensity of electromagnetic energy at each wavelength of light in a specified area [11]. The spectrophotometry method is easy and convenient for obtaining information on the colour distribution by groups of teeth and thirds [14].

In recent years, digital technology has been gaining tremendous recognition in various fields of dentistry [15]. Digital fabrication technology, also referred to as three dimensional (3D) printing, is increasingly applied clinically for dental accessories, such as implant guides, orthodontic power-arms and power-caps, 3D-printed aligners, and splints [16]. The fabrication of removable dentures using additive and subtractive manufacturing facilitated the working protocol of dentists and dental technicians. The processing of the removable dentures became more efficient and profitable, the patient discomfort was reduced, and the quality of the prosthetic restorations was increased [17].

3D-printed dentures are manufactured by the methods of stereolithography (SLA), which includes printing photocurable dental resin with a 3D printer layer by layer. Stereolithography (SLA) is the most common 3D printing process and has the ability to produce high-accuracy prototypes in a range of advanced materials with excellent features [18]. Stereolithography falls under the category of additive manufacturing technologies known as vat photopolymerization. A light source—a laser or a projector—is used to cure liquid resin into hardened plastic. This process depends on the variety of different core components, such as the light source, the build platform, and the resin tank [12].

The STL file format, which represents the geometry of an object in the form of triangles, is standard for transferring three-dimensional information to 3D printers [16]. NextDent Denture 3D + is a biocompatible dental resin used for different elements of removable dentures–denture base and teeth [19]. The physical and mechanical properties of this material are similar to the conventional acrylic resin for removable dentures [20]. Three-dimensional-printed teeth are also manufactured from methacrylate-based photopolymerized resin, which is processed by 3D printing [21]. Denture teeth and denture base are 3D printed separately, and then the printed teeth are attached to the printed denture base using a light-cured bonding agent and a final additional polymerization [22].

The aim of this in vitro study is to investigate and compare the colour stability of NextDent 3D-printed denture teeth and two types of conventional denture teeth in shade A2 after immersion in four staining solutions. The null hypothesis is that there is no significant difference in the colour stability between NextDent 3D-printed denture teeth and conventional teeth for removable dentures.

## 2. Materials and Methods

For this in vitro study, according to ISO 22112/2007, two different types of denture upper central incisors were selected [23]. The first and second groups were prefabricated conventional denture teeth of polymethyl methacrylate used for removable acrylic dentures: Ivostar Shade (Ivoclar Vivadent, Schaan, Liechtenstein) and SpofaDent Plus (SpofaDental, Jičín, Czechia). The third type of artificial central incisors was 3D printed layer by layer, using the method of stereolithography (SLA). The 3D-printed teeth were manufactured by NextDent C&B MFH (NextDent, 3D Systems, Soesterberg, The Netherlands) micro-filled hybrid methacrylate-based photopolymerized resin [10] according to the size and shape of the Ivostar Shade (Ivoclar Vivadent, Schaan, Liechtenstein) prefabricated selected upper central incisor. According to the Vita Classic Shade guide, all denture teeth in the study were from the A2 shade.

**Figure 1:**
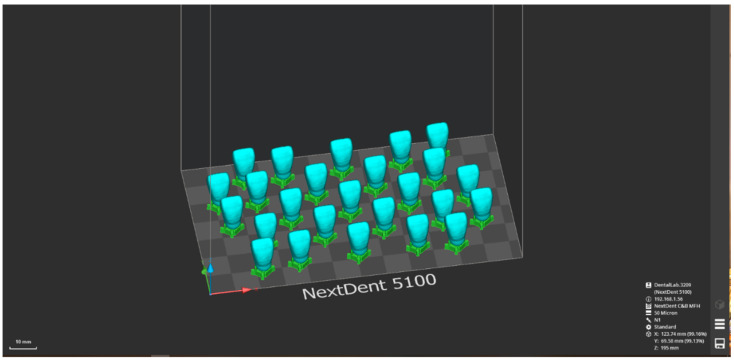
Digital three dimensional (3D) model of the artificial teeth used in this study (NextDent 5100, NextDent, 3D systems, The Netherlands).

A STL file of the digital model was uploaded to the software of the Next Dent 5100 (NextDent, 3D Systems, Soesterberg, The Netherlands) 3D printer, in order to be exported for 3D printing [10]. The artificial upper central incisors were manufactured layer by layer after the dental resin NextDent C&B MFH (NextDent, 3D Systems, Soesterberg, The Netherlands) was placed into the 3D printer (Figure 1).

**Figure 2:**
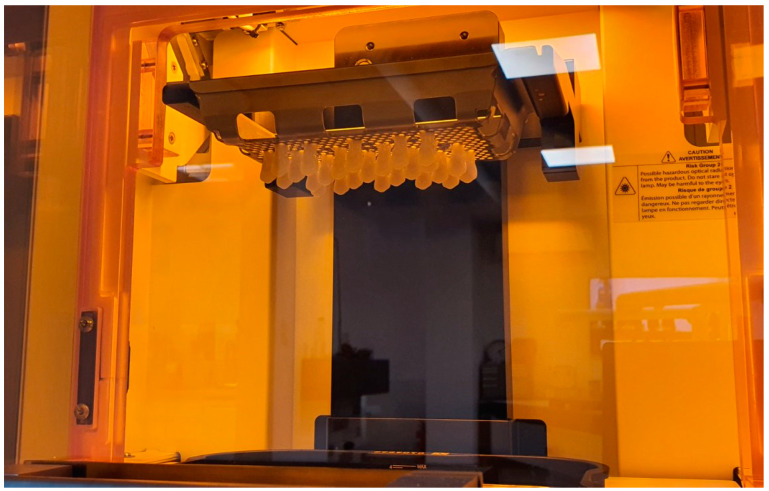
Three-dimensional printing of the artificial teeth: dental resin material NextDent C&B MFH (NextDent 3D Systems, Soesterberg, The Netherlands) is placed into the 3D printer NextDent 5100 (NextDent, 3D Systems, Soesterberg, The Netherlands).

The maxillary central incisors were printed at a 50 µm layer thickness and cleaned with isopropanol. Isopropyl alcohol (IPA—2-propanol or rubbing alcohol) is a clear, powerful cleaning agent used for different 3D-printed materials. IPA is effective in cleaning the 3D printer building board and leaves no traces or residues. For 3D-printed parts, the process usually takes six minutes and the IPA is diluted with distilled water at a ratio of 70% isopropyl alcohol to 30% distilled water [3]. After the cleaning, the specimens were cured for a further 45 min by immersion into glycerin in the post-curing oven to react the remaining monomers (Figure 2).

For the current study the upper central incisors of Ivostar Shade (Ivoclar Vivadent, Schaan, Liechtenstein) and Spofadent™ Plus (SpofaDental, Jičín, Czechia) in shade A2 were selected as conventional types of prefabricated teeth for removable dentures (Table 1).

The specimens were measured by a SpectroShade Micro (SpectroShade, Oxnard, CA, USA) digital spectrophotometer [24] using a piece of dental condensation silicone material (Zetaplus, Zhermack, Badia Polesine, Italy). This silicone mould was prepared for stabilisation of the tooth during measurement and to determinate the shade of each tooth from the same area. During the mould processing, the denture teeth were positioned in a way so that their vestibular surfaces were at the same level as the silicone.

Four types of colourants were used for testing: artificial saliva (AS) as a control group (pH = 6.8), coffee (CF), cola (CO), and red wine (RW). The composition if the artificial saliva is presented in Table 2. 

The artificial teeth (20 in each group, with 5 from each type of denture teeth) were immersed in the selected staining solutions and then stored at 37 °C in four separate glass containers for three time periods: 7, 14, and 21 days. The solutions were replaced every day. The coffee was prepared using 1.8 g of coffee powder (Nescafe Crema, Nestle, Switzerland) in a cup, adding 200 mL of boiling water, then mixing manually for 3 min. The second and third group specimens were stored in glass containers filled with 200 mL cola (Coca-Cola, Coca-Cola Co., Sofia, Bulgaria) and 200 mL red wine (CS Katarzyna, Katarzyna Estate, Bulgaria), respectively. All artificial teeth were cleaned with distilled water before every measurement. The water on the surfaces of the artificial teeth was removed with a paper towel, then the teeth were left to dry.

The colour values of denture teeth were measured with the SpectroShade Micro (SpectroShade, Oxnard, CA, USA) spectrophotometer [24]. The device was calibrated before each measurement according to the manufacturer’s guidelines. The data were recorded using the Commision Internationale d’Eclairage (CIE) *L* * *a* * *b* * colour system [25]. The *L* *, *a* *, and *b* *values of each tooth before (control) and after immersion at the selected time periods (T1, T2, and T3) were determined three times by the same person. The mean values of Δ*L* *, Δ*a* *, and Δ*b* * were automatically calculated via spectrophotometry and recorded. Colour difference (Δ*E* *) was determined from the mean Δ*L* *, Δ*a* *, and Δ*b* * values for each tooth with equation (1). In this formula, Δ*L* *, Δ*a* *, and Δ*b* * are the differences in the *L* *, *a* *, and *b* * coordinates of the colour space: L* (white–black), *a* * (red–green), and *b* * (yellow–blue), respectively [25].

The mean ΔE* value was calculated from the measurements of each tooth after immersion in each of the four types of colouring solution. Average colour changes after 7, 14, and 21 days were also determined. In this current study, the data were analysed using SPSS Statistics 26. The mean and standard deviation values were determined for all teeth groups and staining agents. The colour system selected for this study was L *, a *, and b * of CIE1986. L * a * b * colour difference (Δ*E* *) is the difference between the value of the experimental group after immersion periods and the control group. The value representing the degree of colour difference was multiplied by 0.92 to calculate the National Bureau of Standards value (NBS) (Equation (2)).

NBS unit = Δ*E* × 0.92 (2)

The NBS rating system is used for colour stability determination and shows the human eye’s perceptibility of the colour change [26] (Table 3).

## 3. Results

The mean and standard deviation of colour change (∆*E*) of two types of denture teeth in shade A2, after immersion in four colouring agents for 7, 14, and 21 days, are summarized in Table 4, Table 5, Table 6 and Table 7. The two-way ANOVA analysis determined that the interaction between immersion time and colouring solution was significant at all intervals. This statistical method was applied to assess the interaction effect of type of denture teeth, immersion time, and type of staining agent on the colour stability. The value of ∆*E* for all denture teeth was greatly affected by the immersion time period. The colouring solution, especially combined with the time of immersion, significantly affected the values of ∆E for all types of tested teeth.

After the first week, the value of ∆*E* of all types of tested teeth immersed in red wine showed an almost fourfold increase. During the other two periods (14 and 21 days), a steady increase in the value of ∆*E* was observed. The teeth immersed in coffee also showed a considerable colour change during all three periods, but not as intense as those immersed in red wine. The staining effect of the cola solution was at an acceptable level of colouration at all periods observed, and none of the ∆*E* values surpassed the value of 1. After the first week, there was a slight discolouration effect for the test specimens immersed in artificial saliva. This tendency continued during the second and the third week, but the difference was negligible (Table 4).

Two-way ANOVA analysis was performed to determine whether there is any statistical significance between the value of Δ*E* and the type of material; between the immersion time and the type of staining agent as independent variables; and between their interaction terms (Table 4). The value of the confidence interval used for the test was α = 0.05. In Table 4, the results from the two-way ANOVA are presented. As is clearly seen from the *p*-value column of the table, there is a statistical significance between the value of ∆E and the type of material (*p* < 0.05). The statistical significance between the value of ∆E and the factors of time, staining solution, the interaction of material and staining solution (material × staining solution), and the interaction of time and staining solution (time × staining solution) is especially strong (*p* < 0.001). The *p* value for the interaction of factors time × type of material is equal to 0.125, which is greater than α = 0.05; therefore, the value of Δ*E* is not dependent on the type of material used, nor the immersion time. From this, we can draw the conclusion that time and staining solution used are the two main factors that affected the value of Δ*E*.

Table 4, Table 5, Table 6 and Table 7 present the NBC and SD values. Colour changes of teeth were significantly different in the selected four solutions. The highest value consistently occurred in red wine at all time points, and the type of denture teeth did not affect the degree of staining. The lowest value was observed for AS during all periods of time. Based on the total mean colour changes, the type of staining agent and the duration of immersion were the most important factors influencing the NBC value.

**Figure 3:**
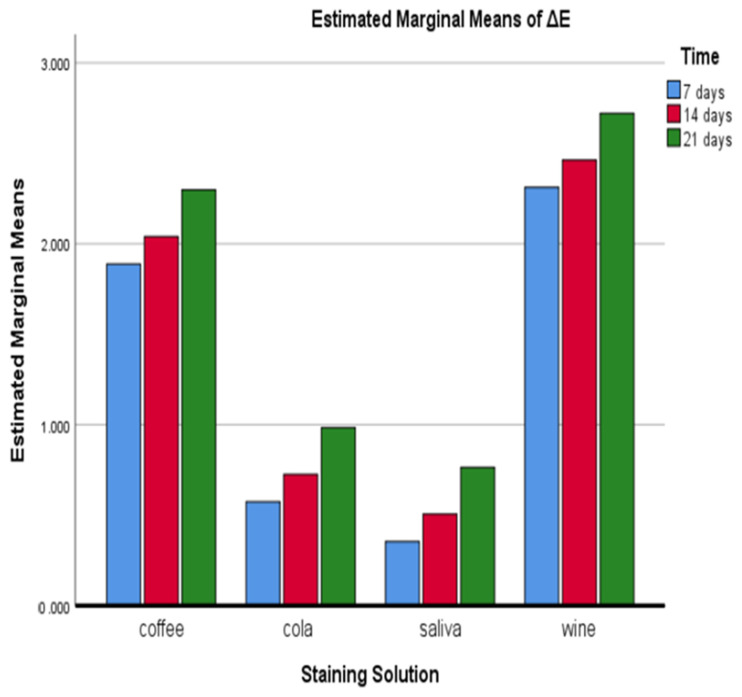
Interactive plot for mean colour difference—interaction between estimated marginal means of ∆*E* and type of staining solution.

The results in Figure 3 represent the interaction between immersion time and colouring agent, which showed the highest and the lowest mean ∆E values for red wine and AS, respectively. The colour changes for red wine, coffee, and cola increased during the three weeks of the observation, and they were most significant after the 21st day. The discolouration effect of AS between the different immersion periods was not significant for the tested denture teeth.

**Figure 4:**
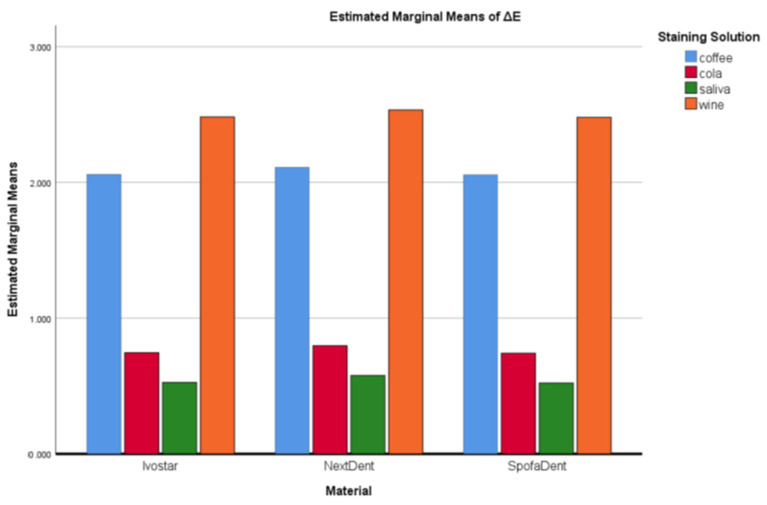
Interactive plot for mean colour difference—interaction between staining solution and type of material.

From the interactions between the type of material and the colouring solutions, it is clear that the highest and the lowest mean ∆*E* values were for SpofaDent Plus and Ivostar in red wine and AS, respectively (Figure 4). For the staining effect of the coffee, the highest value was for NextDent, and the degree of colouration for the other two types of denture teeth was similar. The values for cola were almost the same for all three types of specimens, without any significant difference.

## 4. Discussion

In this in vitro study, three different types of denture teeth, including one brand of 3D-printed and two brands of prefabricated acrylic denture teeth, were immersed in four staining agents. The changes in the colour stability (∆*E*) were measured after 7, 14, and 21 days. The results showed that the different immersion periods influenced the ∆*E* of all denture teeth. Furthermore, the type of selected colouring agent interacted significantly with the different periods of time. Therefore, the null hypothesis that there is no major colour difference in any of the selected teeth irrespective of colouring agent and duration of immersion can be rejected.

Our findings were supported by the studies performed by Moon et al. [27], Tanthanuch et al. [28], and Duymus et al. [29]. In this comparative study, red wine solution showed the highest colour change of all types of denture teeth. All the tested samples showed significant colouration after one week, with a steady increase for the other two periods. This finding is in agreement with that of Ehsani et al. [30]. It was evaluated by the higher polarity of the colourant molecules and the significant percentage of tannins [31].

After three weeks of immersion of the selected teeth groups in cola, Δ*E* showed an acceptable degree of colour change. According to the study by Mousavi et al., colouration in cola is due to the caramel colour, which is made by heating glucose in the presence of acrylic acid [32]. After two weeks of immersion of NextDent and SpofaDent Plus in coffee, an unacceptable colour change occurred. This finding is in agreement with Babikir et al., Alfouzan et al., and Amin et al. [33,34,35].

When immersed in artificial saliva, Δ*E* values at 7, 14, and 21 days showed a slight decrease as time progressed, which is a sign of very light discolouration. The differences among the three values are negligible [36,37,38,39]. According to the research by Koksal and Dikbas, Brewer et al., lighter shades of teeth experience greater discolouration than darker shades [40,41]. Thus, the A2 shade of denture teeth was chosen for our study because it is one of the lightest shades available for the denture teeth brands [42,43,44].

In the current study, the middle third of the vestibular surface of the upper central incisors was investigated for colour changes. This was chosen due to the composition of the artificial teeth in the cervical area, which differs from that in other parts of the crown [45,46].

According to the review of Paolone et al. [9], many researchers investigated dental materials immersed in different colourants. This suggests the necessity for standardization of the method. Moreover, dental resins could be repolished. This could be included in a future investigation, while ∆*E* values could be reverted to clinical acceptability.

Hipolito et al. showed that coffee and cola were the colouring agents which showed the most significant changes in the colour stability of the specimens without any major differences [47]. Catelan et al. [48] proved that all composite resins changed colour after the ageing methods. Furthermore, the surface sealant did not alter the colour stability of the tested materials. Ghahramanloo et al. [49] examined the colour stability of two types of artificial teeth—those manufactured from acrylic and porcelain material—that were immersed in different colouring solutions. Better results were evaluated for the type of porcelain teeth. These studies showed that the denture teeth’s composition and fabrication affect the colour stability [50]. The main reasons for the differences could be related to the type of polymerization, the level of residual monomer, or initiators such as dibenzoyl peroxide [51].

According to the in vitro study of Ehsani et al. [30], the change in colour of all artificial teeth after one month of immersion in three types of colourants (coffee, tea, and cola) was at the acceptable level. All of the artificial teeth were affected significantly by the turmeric solution [52,53]. This research proved that the type of teeth, staining agent, and duration influence the colour stability of the specimens [54].

In this in vitro study, artificial saliva, coffee, red wine, and cola were used as colouring agents. These beverages are widely consumed in many countries. According to Gregorious et al. [55], exposure to coffee is approximately 10 min daily. The results showed that a 1-week exposure to the coffee solution corresponds to approximately 34–67 months of constant intake, which results in colour changes.

## 5. Conclusions

The findings of this in vitro study determined that 3D-printed teeth showed better colour stability than PMMA denture teeth, although the differences were not significant. Therefore, denture teeth manufactured by the method of 3D printing could be a sensible choice for manufacturing a removable denture, which will expand the material list for treatment options. However, all Δ*E* values were below 3.3, which indicated that all tested teeth were clinically acceptable and the highest values for colouring were for red wine and the immersion period of 21 days.

## Figures and Tables

**Table 1:** Types of denture teeth used in this study.

Product	Composition	Detail	Manufacturer
NextDent	Methacrylate-based photopolymerized resin	3D printing	3D Systems, Soesterberg, The Netherlands
Ivostar Shade	MPM–PMMA *	Mold XS	Ivoclar Vivadent, Schaan, Liechtenstein
Spofadent™Plus	MPM–PMMA *	Mold XS	SpofaDental, Jičín, Czechia

* MPM—multiplex polymer matrix; PMMA—polymethylmethacrylate.

**Table 2:** Composition of the artificial saliva, used in this study.

Component	%
NaCl	0.0856
KCl	0.1200
MgCl_2_ × 6H_2_O	0.0052
Mannitol	0.2000
K_2_HPO_4_	0.0456
Carbomer 974P	0.1000
NaOH 10%	0.4000
CaCl_2_ × 2H_2_O	0.0148
KH_2_PO_4_	0.0272
Purified Water	96.9016

**Table 3:** National Bureau of Standards (NBS) ratings.

NBC Unit	Critical Remarks of Colour Differences	
0.0–0.5	Trace	Extremely slight change
0.5–1.5	Slight	Slight change
1.5–3.0	Noticeable	Perceivable change
3.0–6.0	Appreciable	Marked change
6.0–12.0	Much	Extremely marked change
12.0 or more	Very much	Change to other colour

**Table 4:** Colour changes over time in different types of denture teeth (Δ*E*).

Type of Material	Period of Time (T)	Artificial Saliva Δ*E* (Mean)	Red Wine Δ*E* (Mean)	Coffee Δ*E* (Mean)	Cola Δ*E* (Mean)
NextDent Ivostar Shade SpofaDent Plus	7 days (T1)	0.587 0.548 0.562	2.050 2.194 2.185	1.829 1.702 1.725	0.658 0.676 0.667
NextDent Ivostar Shade SpofaDent Plus	14 days (T2)	0.556 0.529 0.535	2.458 2.495 2.498	2.174 1.834 1.876	0.765 0.746 0.728
NextDent Ivostar Shade SpofaDent Plus	21 days (T3)	0.528 0.509 0.514	2.832 2.876 2.898	2.728 2.459 2.350	0.884 0.867 0.854

**Table 5:** Two-way ANOVA for Δ*E*.

Label	Sum of Squares	DF	Mean Square	F	*p*-Value
Type of Material	0.023	2	0.011	7.732	0.007
Time	1.030	2	0.515	353.790	*p* < 0.001
Staining Solution	25.115	3	8.372	5749.859	*p* < 0.001
Material × Staining Solution	0.119	6	0.020	13.674	*p* < 0.001
Material × Time	0.013	4	0.003	2.247	0.125
Time × Staining Solution	0.751	6	0.125	86.019	*p* < 0.001
Error	0.017	12	0.001	0.00	0.00

**Table 6:** Colour changes over time in different types of denture teeth (NBC Unit).

Type of Material	Period of Time (T)	Artificial Saliva (NBC Unit)	Red Wine (NBC Unit)	Coffee (NBC Unit)	Cola (NBC Unit)
NextDent Ivostar Shade SpofaDent Plus	7 days (T1)	0.540 0.504 0.517	1.886 2.018 2.010	1.683 1566 1.587	0.605 0.622 0.614
NextDent Ivostar Shade SpofaDent Plus	14 days (T2)	0.512 0.487 0.492	2.261 2.295 2.298	2.000 1.687 1.726	0.704 0.686 0.670
NextDent Ivostar Shade SpofaDent Plus	21 days (T3)	0.486 0.468 0.473	2.605 2.646 2.666	2.510 2.262 2.162	0.813 0.798 0.786

**Table 7:** Colour changes over time in different types of denture teeth. **(Standard Deviation—SD)**.

Material	Staining Solution	Mean	N	Std. Deviation (SD)	Minimum	Maximum
Ivostar Shade	coffee	1.99833	3	0.404372	1.702	2.459
	cola	0.76300	3	0.096628	0.676	0.867
	saliva	0.52867	3	0.019502	0.509	0.548
	wine	2.52167	3	0.341781	2.194	2.876
	Total	1.45292	12	0.898983	0.509	2.876
NextDent	coffee	2.24367	3	0.453531	1.829	2.728
	cola	0.76900	3	0.113053	0.658	0.884
	saliva	0.55700	3	0.029513	0.528	0.587
	wine	2.44667	3	0.391123	2.050	2.832
	Total	1.50408	12	0.922591	0.528	2.832
SpofaDent Plus	coffee	1.98367	3	0.326114	1.725	2.350
	cola	0.74967	3	0.095364	0.667	0.854
	saliva	0.53700	3	0.024062	0.514	0.562
	wine	2.52700	3	0.357384	2.185	2.898
	Total	1.44933	12	0.894111	0.514	2.898

## Data Availability

Not applicable.
